# Spatial extent of dysbiosis in the branching coral *Pocillopora damicornis* during an acute disease outbreak

**DOI:** 10.1038/s41598-023-43490-3

**Published:** 2023-10-02

**Authors:** Austin Greene, Tess Moriarty, William Leggatt, Tracy D. Ainsworth, Megan J. Donahue, Laurie Raymundo

**Affiliations:** 1 University of Hawai‘i at Mānoa, Honolulu, USA; 2 Hawai‘i Institute of Marine Biology, Kāne‘Ohe, HI USA; 3 Woods Hole Oceanographic Institution, Woods Hole, USA; 4 University of Newcastle, Ourimbah, Australia; 5 University of New South Wales, Sydney, Australia; 6 University of Guam Marine Laboratory, Guam, USA

**Keywords:** Ecology, Ecological epidemiology, Microbial ecology

## Abstract

Globally, coral reefs face increasing disease prevalence and large-scale outbreak events. These outbreaks offer insights into microbial and functional patterns of coral disease, including early indicators of disease that may be present in visually-healthy tissues. Outbreak events also allow investigation of how reef-building corals, typically colonial organisms, respond to disease. We studied *Pocillopora damicornis* during an acute tissue loss disease outbreak on Guam to determine whether dysbiosis was present in visually-healthy tissues ahead of advancing disease lesions. These data reveal that coral fragments with visual evidence of disease are expectedly dysbiotic with high microbial and metabolomic variability. However, visually-healthy tissues from the same colonies lacked dysbiosis, suggesting disease containment near the affected area. These results challenge the idea of using broad dysbiosis as a pre-visual disease indicator and prompt reevaluation of disease assessment in colonial organisms such as reef-building corals.

## Introduction

Climate-related impacts and human interventions to restore ecosystems are becoming more common. Coral reefs have severely declined, in part due to disease outbreaks caused by thermal stress and degraded water quality ^1–3^. The ongoing spread of Stony Coral Tissue Loss Disease (SCTLD) underscores an urgent need for effective management strategies as disease events reshape coral reef habitats ^4^. There is a critical need to develop tools for diagnosing, treating, and managing coral disease, particularly to reduce colony mortality and preserve live coral cover. Doing so will require a deep understanding of the complex colonial coral meta-organism ^5,6^.

The coral meta-organism includes a long-lived colonial coral animal which grows through the asexual reproduction of individual coral polyps to form a colony, a functionally crucial endosymbiosis with photosynthetic dinoflagellates for energy production, and a diverse microbiome found in all coral tissue compartments ^7,8^. This microbiome plays a fundamental roles in host fitness ^9^, biogeochemical cycling ^10^, and long-term persistence of coral reefs ^11^. Recent research shows coral microbial communities change in response to disease, impacting microbial composition ^9,12–16^ as well as function reflected in altered metabolite production ^17^. Consequently, coral microbiomes and metabolomes are now key indicators of coral health states, including disease ^18,19^. With increasing utilization of these tools comes a need to better understand and validate the scales at which microbial structure and metabolomic function of the coral holobiont vary under disease conditions.

While corals often exhibit colony-wide stress responses, such as thermal bleaching, these do not rule out alternative responses that are constrained to one area of these structurally complex, colonial organisms. When coral diseases occur, a multitude of factors can contribute to coral tissue pathologies differing across the surface of a colony. For example, perforate corals feature tissue compartments rooted in the underlying skeleton and polyps are substantially more interconnected compared to species with an imperforate skeletal strategy. Light intensity varies across and within the coral colony ^20^ and coincides with microbial diversification among coral compartments ^21^. Somatic mutations accrue as corals grow, providing a genetic basis for intra-colony functional differentiation ^22^. Furthermore, polyp interconnectivity is modulated by complex, spatially-constrained surface flows ^23^ that may explain how surface mucus microbiomes differ at small scales in some coral species ^24^ or why coral surface fluorescence becomes fragmented during disease ^25^. The treatment of diseases such as SCTLD ^26^ or Black Band Disease ^27^ with antimicrobial pastes applied at the lesion margin, while varied in efficacy ^28^, provides compelling evidence that disease may not wholly compromise coral colonies. In light of this evidence and the colonial life strategy of many reef-building corals we hypothesized that the effects and indicators of disease would vary across a coral colony's surface.

Studies often search for disease indicators by comparing the microbiomes of coral colonies labeled as diseased or healthy. While some efforts have been at least temporarily successful in identifying causative pathogens ^29^ with ASV-level analysis, there is a growing recognition that many coral diseases arise from polymicrobial consortia, reducing the value of any single taxa as an indicator. For example, while progression of Black Band Disease is dependent on the interplay of filamentous cyanobacteria, sulfate-reducing bacteria, and sulfide-oxidizing bacteria, the identity of these functional groups varies by region ^30^. Many studies now focus on identifying broad microbial differences between coral health states, following the Anna Karenina Principle (AKP), which suggests greater microbial composition variability among unhealthy individuals than healthy ones ^31^—a phenomenon otherwise known as “dysbiosis”. Here, we define dysbiosis as a statistically significant difference in the overall microbial composition of visually-healthy and diseased tissues. Importantly, for dysbiosis to be a reliable early (e.g. pre-visual) indicator of coral health ^32^, it necessitates a colony-wide response to disturbance. The colonial nature of many reef-building corals, along with genetic mutations, microenvironments, and baseline microbial variability, may hinder dysbiosis as a reliable early indicator of coral disease.

**Figure 1:**
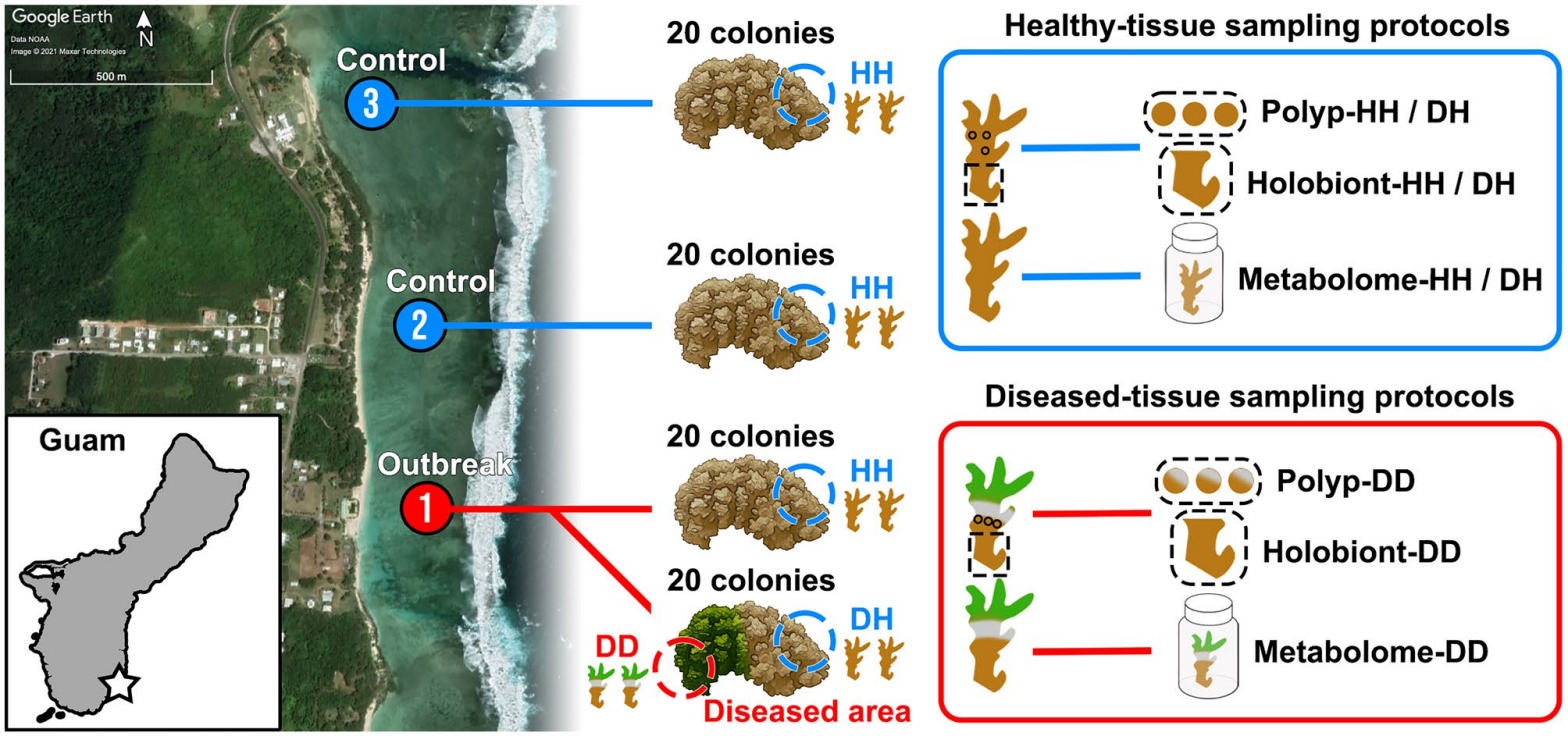
Disease-outbreak and healthy-control sampling locations located in Southeast Guam and the sampling scheme deployed at each location for either healthy or diseased coral colonies. Visually-healthy fragments were collected from control healthy *Pocillopora damicornis* colonies (HH) as well as from unaffected regions of diseased colonies (DH). Fragments with active lesions were also collected from disease-affected colonies (DD). Fragments from each tissue type underwent polyp-scale biopsies and larger tissue extractions for microbial content, as well as whole-fragment methanol extraction for metabolomic content. On fragments with visual evidence of disease (DD) three intact polyps were biopsied near the lesion front, and later pooled, while a larger holobiont sample was collected just behind this area approximately 1 cm from the lesion.

*Pocillopora damicornis* is a common, finely-branching coral found in shallow reef environments, known for its adaptability and often considered a weedy species. Over a ten-year monitoring program at six inshore reefs in Guam, baseline prevalence of slow-progressing tissue loss diseases affecting *P. damicornis* was documented at 3.8%, with no known historical outbreaks (Raymundo unpublished data). In September 2018, a rapid-progressing tissue loss syndrome was observed on multiple colonies of *P. damicornis* in Tanguisson, a shallow reef flat on the northwestern coast of Guam resulting in a mean prevalence 15.5%. By December, prevalence at this site had risen to 17.9%. In early November 2018, the same tissue loss pattern was observed among *P. damicornis* (17 cm diameter average) on a nearshore reef flat along the southeastern coast of Guam (Fig. 1, outbreak site) with an estimated prevalence of more than 20%. The tissue loss disease manifested as a single, discrete ovoid area of tissue loss beginning on the top of the colony and progressing from branch tips downward, resulting in complete colony mortality within one to two weeks*.*

To assess the utility of dysbiosis as disease indicator we examined those tissues most likely to display early signs of impact: visually-healthy tissues on *P. damicornis* colonies with active disease lesions. Briefly, we compared the microbiome of tissues immediately ahead of disease lesions to those collected in a visually healthy area of the same colony, as well as to visually-healthy colonies from two control reef sites. To address if sampling methods affect detection of dysbiosis, these comparisons were repeated for large “holobiont” samples that included multiple polyps with connective tissue and skeleton, and for a subset of samples using pooled biopsies of three single polyps without connective tissue or skeleton. Metabolomic comparisons were completed among a subset of samples to investigate if dysbiosis also presents in the coral metabolome as a functional impact of disease. Our findings show that coral pathologies can be localized within a colony and dysbiosis is not guaranteed, even in tissues near active disease lesions with impending complete colony mortality.

## Results

### Sequencing results

A total of 87 holobiont samples and 70 polyp biopsy samples remained after quality filtering of microbial samples. Eighty six suspected contaminant ASVs were identified and removed from the holobiont dataset (0.58% of total ASVs), compared to 17 in the polyp dataset (0.08% of total ASVs). Holobiont samples featured an average read depth of 75,222 compared to 10,743 in negative controls for this sample type and were subsequently rarefied to a read depth of 30,000 resulting in the loss of eight samples. Polyp biopsy samples featured an average read depth of 43,804 compared to 8893 for negative controls and were rarefied to a read depth of 10,000 resulting in the loss of seven samples. Summaries of sequencing read depth data holobiont and polyp samples are available in Table S1.

### No dysbiosis in healthy tissues from diseased colonies

Microbial dissimilarity was greatest in diseased tissues from diseased colonies (DD) and reduced in visually-healthy tissues from healthy (HH) or diseased (DH) colonies. For holobiont samples, mean Bray–Curtis dissimilarity was 81.5% for samples adjacent to disease lesions (Holobiont-DD), 64.3% for healthy tissue from diseased colonies (Holobiont-DH), and 60.1% for healthy tissue from control colonies (Holobiont-HH); for polyp samples, intersample dissimilarity was 87.8% at the lesion front (Polyp-DD), 71.5% for healthy tissue on diseased colonies (Polyp-DH), and 80.2% for healthy tissue on healthy colonies (Polyp-HH). ANOSIM tests indicated no significant microbial dissimilarity between healthy tissues from visually-healthy coral colonies taken at any site for either holobiont or polyp samples (Fig. S1).

**Figure 2:**
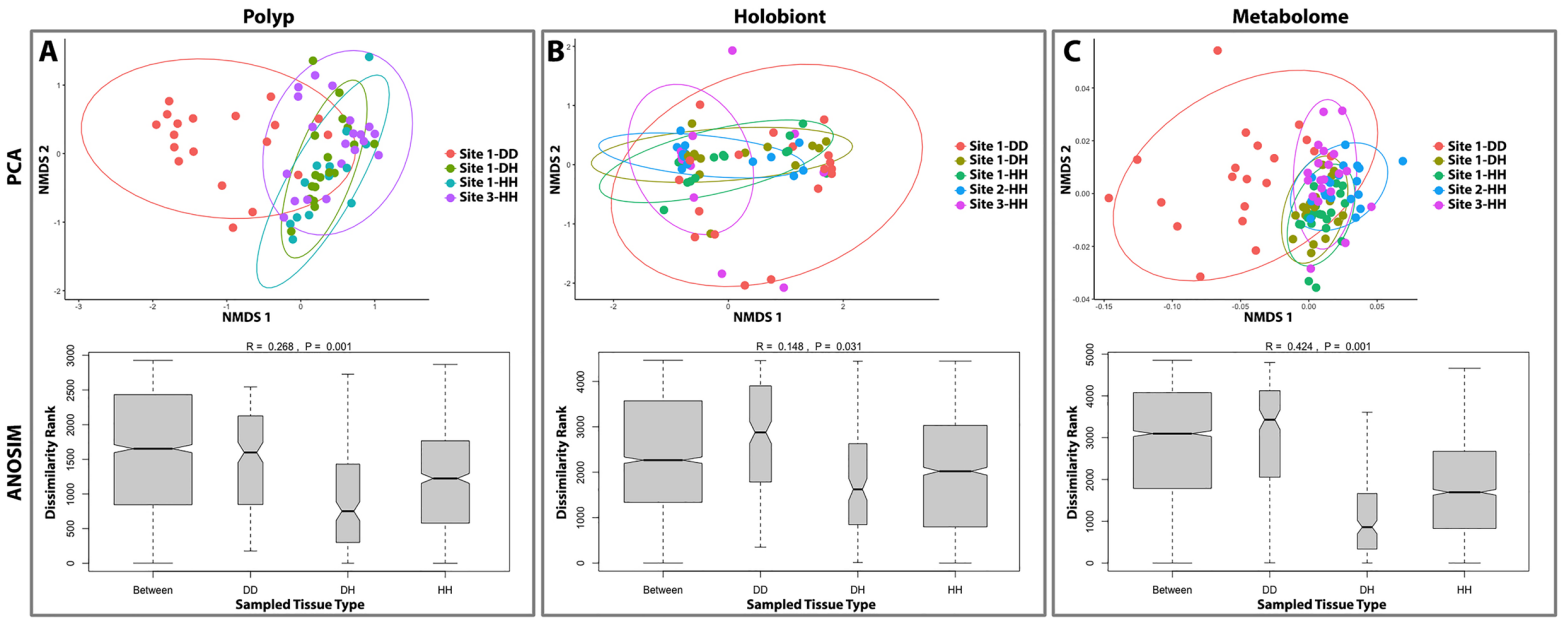
Principal coordinate analysis (PCA, above) and ANOSIM results (below) comparing dissimilarity among diseased tissue on diseased colonies (DD), visually-healthy tissue on diseased colonies (DH), and healthy tissue on healthy colonies (HH) for (**A**) polyp microbial biopsies, (**B**) holobiont microbial samples, and (**C**) whole-fragment metabolomics. ANOSIM results show pairwise distances between all samples, DD samples (outbreak site only), DH samples (outbreak site only) and HH samples (all three sites) highlighting the higher dissimilarity among DD samples and comparatively low dissimilarity among DH or HH samples. Note that bar width indicates the number of pairwise comparisons in each sample subset.

DD samples were significantly different in microbial composition from DH and HH samples. PERMANOVA tests of samples from the outbreak site indicated that microbial dissimilarity varied significantly with tissue type (DD, DH, HH) within both the holobiont and polyp biopsy sample sets (PERMANOVA, Fig. 2A,B, Table 1). Subsequent tests demonstrated that holobiont samples ~1 cm from the lesion front (Holobiont-DD) were microbially distinct from healthy tissues of the same colony taken away from the lesion (Holobiont-DH), and this pattern was retained in the polyp biopsy sample set (Table 1). In contrast, microbial community composition was not significantly different between healthy tissues on diseased colonies (Holobiont-DH or Polyp-DH) and those sampled from visually-healthy colonies at the outbreak or control sites (Holobiont-HH or Polyp-HH, Table 1). ANOSIM tests confirmed that microbial dysbiosis varied significantly and consistently with tissue type regardless of holobiont or polyp biopsy sampling methods used (Fig. 2). In each of these comparisons, microbiome dissimilarity was at a maximum among DD tissues and a minimum in DH tissues compared to HH tissues from healthy control colonies.

**Table 1:** Results of PERMANOVA analyses of the coral microbiome detected through holobiont and polyp sampling protocols, across different tissue types. Microbiome of fragments with visual evidence of disease (DD) differed from healthy fragments of the same colony (DH), from healthy fragments of healthy colonies (HH) at the outbreak site, and from healthy fragments from health colonies (HH) at all sites. PERMANOVA tests indicate no significant difference in overall microbiome composition between healthy tissues on diseased colonies (DH) and healthy tissues from control colonies at the outbreak site or two control sites (HH).

Comparison	Sample type	Sites included	P	R2	F
DD vs DH vs HH	Holobiont	Outbreak site only	0.02	0.07	2.03
DD vs DH vs HH	Polyp	Outbreak site only	0.001	0.14	2.42
DD vs DH	Holobiont	Outbreak and control sites (S2, S3)	0.03	0.06	2.26
DD vs DH	Polyp	Outbreak and control site (S3)	0.001	0.16	2.53
DH vs HH	Holobiont	Outbreak site only	0.49	0.02	0.80
DH vs HH	Polyp	Outbreak site only	0.42	0.07	1.02
DH vs HH	Holobiont	Outbreak and control sites (S2, S3)	0.27	0.02	1.14
DH vs HH	Polyp	Outbreak and control site (S3)	0.32	0.05	1.08

### Limited metabolomic dissimilarity in lesion and lesion-adjacent tissues

Beta dispersion of coral metabolome samples differed significantly among tissue types (df = 2, F = 12.61, p = 0.001). ANOSIM analysis revealed that metabolomic dissimilarity varied significantly among tissue types (R = 0.42, p = 0.001) and was at a maximum in diseased tissues (DD, Fig. 2C). Variability in metabolome composition closely followed patterns observed in the microbiome: dissimilarity was greatest among fragments with lesion tissue (DD) compared to fragments with visually-healthy tissue from the same colony (DH) or fragments from healthy control colonies (HH). At the outbreak site, there was no significant dissimilarity between the metabolome of visually-healthy fragments from diseased colonies (DH) and fragments from healthy control colonies (HH). Across sites, PERMANOVA tests determined that fragments from visually-healthy coral colonies (HH) varied in their metabolome based on the site they originated from (Fig. S3), this effect was significant at the time of the outbreak (df = 2, R2 = 0.09, F = 5.03, Pr(> F) = 0.001) and in a set of HH fragments sampled haphazardly one year after the outbreak (df = 2, R2 = 0.36, F = 1.9, Pr(> F) = 0.01). Year 2 metabolome data were not included in any other analysis following detection of consistent site-related metabolome differences.

### Characterizing a microbial shift at the lesion front

Comparisons of microbial composition in paired DD-DH samples taken from the same diseased colony identified amplicon sequence variants (ASVs) that differed between DD and DH tissue types in either the holobiont or polyp biopsy sample set. Among paired holobiont DD-DH samples, one unclassified Cyanobacteria was significantly less abundant in DH samples compared to DD samples (p = 1.06e−7, FDR = 6.17e−6, logFC = − 6.24). Ten ASVs were detected as occurring differently between polyp DD and DH samples. Four ASVs, all classified as *Endozoicomonaceae* (order *Oceanospirillales*) were elevated in healthy tissues from diseased colonies (Polyp-DH) compared to diseased tissues from the same colony. Six ASVs were at elevated abundance in polyps at the lesion front (Polyp-DD) compared to than healthy tissues on the same colony (Polyp-DH). These disease-associated taxa included two *Erythrobacteraceae* (order *Sphingomonadales*), two *Xenococcaceae* (order *Chroococcales*), and one unclassified ASV from order *Alteromonadales.*

**Figure 3:**
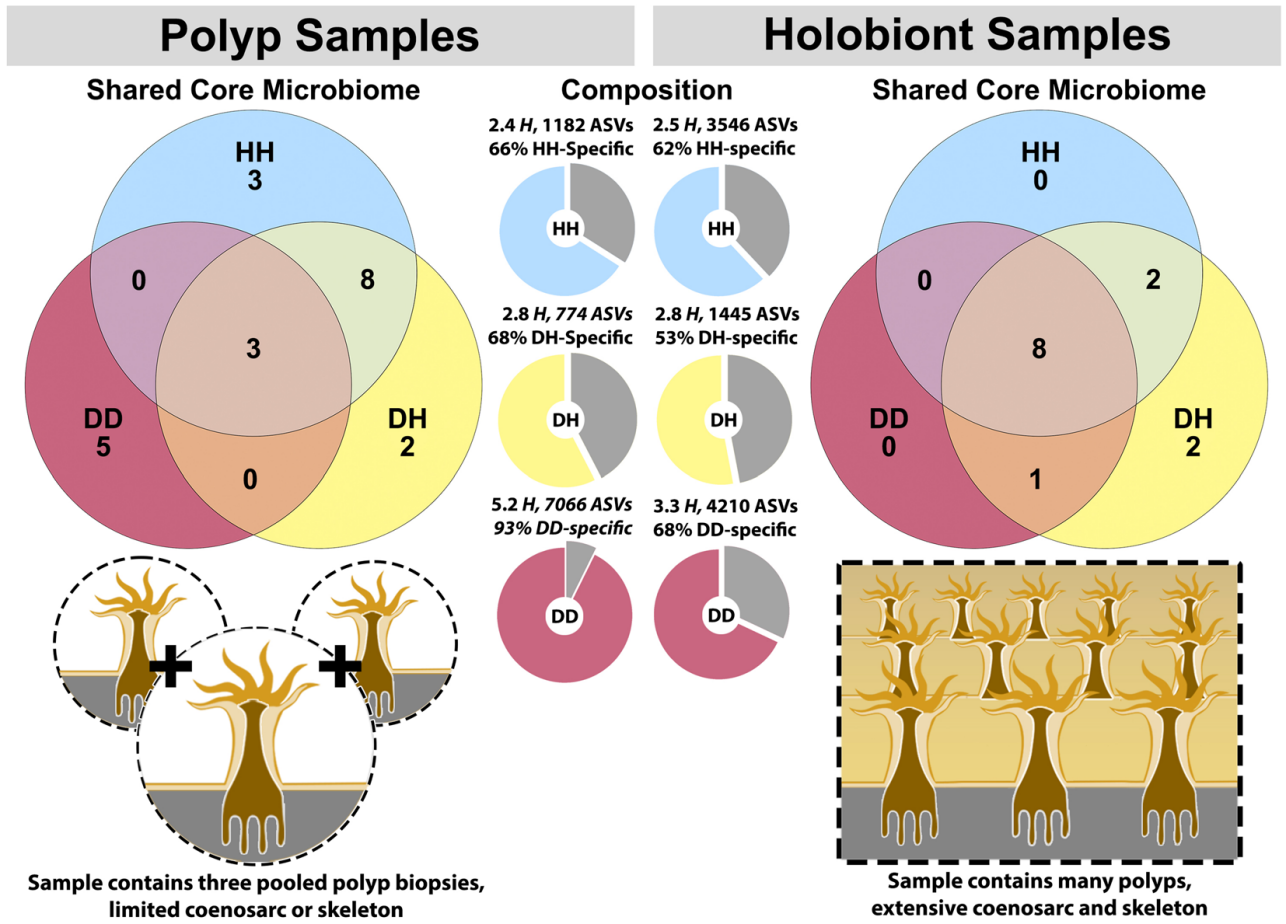
Consistent patterns of microbiome composition observed between targeted polyp biopsies (3 × 1 mm polyps per sample) and holobiont samples (approx. 1 cm^2^) containing a mixture of polyps, coenosarc, and skeleton. Greater overlap in high-prevalence (≥ 75%) “core microbiome” ASVs occurs between healthy tissues on diseased colonies (DH) and healthy tissues on control colonies (HH) than with diseased tissues on diseased colonies (DD). Similarly, though technique and sequencing effort differ substantially between polyp and holobiont methods (30,000 vs 10,000 reads), both methods detect that microbiome specificity is highest at or near the lesion front (DD) with taxa that do not occur in any other tissue type (DH or HH).

In both the polyp biopsy and holobiont sample sets microbial communities adjacent to the disease lesion (DD) had greater diversity and more taxa unique to this tissue type than healthy tissues from diseased (DH) or healthy colonies (HH). Wilcoxon rank sum tests indicated that Shannon microbial diversity was significantly higher in polyps at the lesion front (5.2 *H,* Polyp-DD) and holobiont samples near the lesion (3.3 *H,* Holobiont-DD) compared to healthy tissues from diseased colonies (DH) or control healthy colonies (HH, Fig. 3). The full results of these Wilcoxon rank sum tests comparing microbial communities across all tissue types within each sampling method is available in Tables S2 and S3. Diseased tissues (DD) also demonstrated a high number of taxa that did not occur in healthy tissues (DH or HH). On average 93% of ASVs in Polyp-DD samples were found only in Polyp-DD samples, and 68% of ASVs in Holobiont-DD samples were only found in other Holobiont-DD samples (Fig. 3).

The core microbiome was defined as ASVs present in at least 75% of unrarefied samples. Using this criteria we detected 21 core ASVs in polyp samples and 13 core ASVs in holobiont samples, representative sequences and taxonomy of these ASVs are available in Supplementary Data 1 and 2. Of the 21 ASVs in the polyp core microbiome, eight were shared between HH and DH tissue types, three were shared by all polyp tissue types (DD, DH, HH), and no high-prevalence core ASVs were shared between polyp DD and DH or HH. Five core ASVs were unique to DD polyps sampled at the lesion front (Fig. 3, left panel). Of 13 ASVs in the holobiont core microbiome, eight ASVs were shared by all tissue types, two ASVs were shared by DH and HH, one ASV was shared by DD and DH, and no core ASVs were shared by DD and HH (Fig. 3, right panel).

In both holobiont and polyp samples from diseased colonies we observed a similar pattern of elevated *Endozoicomonaceae* abundance among visually-healthy tissues (DH) and greatly reduced abundance of this group at the lesion front (Polyp-DD) or approximately 1 cm away from the lesion (Holobiont-DD, Fig. S2). The opposite was observed for *Alphaproteobacteria*, specifically *Rhodobacteraceae,* which were greatly increased in abundance at or near the lesion front compared to healthy tissues from the same diseased colonies (Fig. S2).

## Discussion

Using microbial or metabolic dysbiosis as a pre-visual indicator relies on the presence of a colony-wide response to disease, which was not observed in our study across three different sampling techniques. The coral microbiome is often considered an indicator of colony health and environmental impact ^33^ and dysbiosis is regularly cited as a diagnostic of coral health ^6,13,31,32,34,35^. Dysbiosis, defined here as a significant difference in microbial or metabolomic composition between visually-diseased and visually-healthy coral tissues, was not observed in visually-healthy tissues on colonies affected by disease. This finding held true for both small-scale polyp tissues, larger holobiont samples encompassing multiple coral compartments (coenosarc, polyps, skeleton), and whole-fragment metabolomic analyses. Thus, dysbiosis alone may not reliably indicate coral disease in the absence of visual signs or other markers.

We found that dysbiosis in the coral microbiome was limited to visually diseased fragments. Comparative analysis between lesions and visually healthy tissues on the same colony revealed disease-related taxonomic shifts. In lesions, there was a significant decrease (approximately 70%) in the relative abundance of *Endozoicomonaceae*, a proposed bacterial symbiont of Pocilliporid corals ^4,16^, compared to polyps from healthy tissues on the same colony (DD vs DH, Figure S2). Polyps near lesions exhibited an overabundance of taxa from *Erythrobacteraceae*, *Xenococcaceae*, and *Alteromonadales*, the latter of which have been linked to coral diseases including SCTLD ^32^ and Black Band Disease ^36^, and are considered antagonistic ^37^. The loss of *Endozoicomonaceae* in coral tissues following stress is well-documented ^38^, often followed by an increase in *Rhodobacteraceae* ^39^, as was observed near the disease margin in our samples (Fig. S2). Differences in disease-indicative ASVs between polyp and holobiont samples could be due to methodological variations, different microbes associated with polyps versus other holobiont tissues, or slight proximity differences to the disease lesion (~ 1 cm) of these sampling methods. Identifying specific ASVs or bacterial strains as disease indicators is often challenging, especially in polymicrobial coral diseases or those characterized by opportunistic taxa ^15^. Even in well-studied coral diseases such as White Pox Disease, where established pathogens have previously satisfied Koch’s Postulates, disease etiology can shift over time, and visual indicators may not align with microbial indicators ^29^. Instead, we recommend focusing on the functional roles of taxa associated with, or lost to, disease and their contribution to primary, secondary, or tertiary pathogenesis ^6^.

Visually-healthy tissues on diseased corals (DH) showed no significant differences in microbial or metabolomic content compared to tissues on healthy colonies at the outbreak site. While one interpretation could suggest that apparently healthy colonies were actually diseased during sampling, subsequent follow-up surveys one year post-outbreak indicated this was unlikely, as unaffected corals remained while diseased colonies suffered complete mortality (Raymundo pers. obs.). Metabolomics, which has been used to characterize coral bleaching ^40^ and Stony Coral Tissue Loss Disease ^1^, remains underutilized in the study of most coral diseases. Metabolomic analysis of whole coral fragments supported microbial findings, revealing significant metabolomic differences only in fragments with visible disease signs compared to healthy controls. Therefore, neither microbial nor metabolomic dysbiosis reliably indicated disease in fragments or tissue samples lacking visible signs of the condition.

We found that dysbiosis, whether microbial or metabolomic, is not consistently present throughout the surface of diseased corals. This aligns with a growing body of research suggesting that reef-building corals, as colonial organisms, experience disease in complex ways different from human medicine's conventional techniques and assumptions that have influenced the study of coral disease. Prior research on *Acropora hyacinthus* with White Syndromes showed that apparently-healthy tissues on diseased corals had similar gene expression to healthy controls, with only slight immune changes ^41^. Similarly, transcriptomic and microbiome analysis of Yellow Band Disease revealed that healthy tissues on diseased colonies differ from both healthy controls and diseased tissues ^42^. Confocal microscopy of *Montipora capitata* demonstrated that fluorescent pigments in surface tissues became fragmented and patchy under disease, indicating a breakdown in tissue integration ^25^, potentially hindering disease detection elsewhere on the colony. In the Caribbean, a study tracking individual colonies before and after partial disease mortality found no long-term microbial composition changes, and like our work, visually-healthy tissues on diseased colonies resembled healthy controls ^43^. The complex physical structures of corals, such as the intricate branching of *P. damicornis*, create diverse microenvironments ^20,44^, contributing to microbial differences across the colony, possibly through surface currents ^23^. Additionally, genetic differentiation occurs across the surface of reef-building corals due to somatic mutations ^22^. These studies, and our result of spatially-constrained dysbiosis, lend support to the hypothesis that limited polyp interconnectivity acts as a boundary to disease progression.

Alternatively, these polyps with limited immune response ^41^ and naïve to an adjacent lesion may actually promote disease progression by preventing a colony-wide response. Both hypotheses are possible in the absence of colony-wide dysbiosis and pose essential questions for further coral disease research.

That diseased corals, even those with visible lesions, may not be entirely compromised has significant implications for early disease detection and mitigation strategies that aim to preserve intact coral tissues ^45,46^. Antibiotic and antimicrobial pastes have been widely used to combat coral disease with varying success. For example, in Hawaii, chlorinated epoxy halted an outbreak Black Band Disease ^27^. However, chlorinated epoxy has generally proved less effective for the treatment of SCTLD compared to amoxicillin ^47^. Treatment failures are usually characterized as continued lesion progression ^28^ or reinfection of the coral following treatment and our results provide context for both scenarios. Polyp biopsies in this study demonstrated that coral microbiomes at the lesion front are compromised and holobiont samples occurring ~ 1 cm away from the lesion also exhibit microbial and metabolomic dysbiosis—this distance may inversely scale with morphological complexity. We hypothesize that antibiotic applications would be most effective if made further away from disease margins to fully capture affected tissues. Similarly, we hypothesize that new disease lesions appearing on previously treated corals, but not in areas of prior necrosis, represent the infection of coral tissues naïve to prior infection, rather than a colony-wide “reinfection” event. While antibiotic treatments are scrutinized for their effectiveness and potential impact on the coral holobiont ^48^, studies such as ours can inform the development of new disease treatments.

Coral disease has tripled in prevalence over the past 25 years of climate change ^49^. Acute outbreaks like those studied here leave little time for management action. The biological mechanisms of coral disease remain an area of study rich with uncertainty and complicated by the remarkable complexity of colonial reef-building corals. While some large-scale impacts such as thermal stress lead to consistent colony-wide responses ^40^, this cannot be assumed. Our study shows that microbial and metabolomic dysbiosis are not colony-wide responses to disease in *P. damicornis.* Similar patterns have been observed in other coral species including colonial *Octocorallia* ^50^, which diverged from *Hexacorallia* hundreds of millions of years ago ^51^. Our results add nuance to the understanding of coral disease, shift expectations away from colony-wide responses, and suggest that colonial life strategies may play a major role in coral resilience as disease grows more common under a changing climate.

## Methods

### Sample collection

In early 2018, sampling materials were prepared in anticipation of coral disease outbreaks on Guam's reefs ^52^. In September of the same year, a rapid tissue loss syndrome was observed on *P. damicornis* colonies on the northwestern coast of Guam near Tanguisson during routine monitoring. By early November 2018, the same tissue loss pattern was observed among numerous colonies on a southeastern reef flat along (Fig. 1, outbreak site) dominated by *P. damicornis.* Sampling occurred at this outbreak site on 13 November 2018 and at two control sites located 500 m and 1 km north of the outbreak location.

At the outbreak site, a total of 40 colonies were sampled (20 visually-healthy, 20 visually-diseased), and an additional 20 visually-healthy colonies were sampled from each control site (Fig. 1). Diseased colonies at the outbreak site yielded four fragments each: two from the lesion margin (DD) and two from distant healthy tissue on the same colony (DH). Healthy colonies from outbreak and control sites were sampled by collecting two 4 cm fragments from visually-healthy tissues (HH). Source colonies were photographed and fragments transported on ice to the University of Guam Marine Laboratory (UOGML). At UOGML, one fragment from each sample was stored in methanol for metabolomic analysis, and the second fragment was fixed in 4% paraformaldehyde followed by storage in phosphate-buffered saline for microbiome analysis—fixation in PFA was used to enable future histological analysis not included in this manuscript. These tissue samples were used to investigate the coral microbiome and metabolome of healthy tissues on healthy colonies (HH), healthy tissues on diseased colonies (DH), and diseased tissues on diseased colonies (DD) (Fig. 1). Fragments used for microbial analysis were further subsampled using two different techniques to determine if dysbiosis was detectable in samples containing many polyps plus connective tissue and underlying skeleton (“Holobiont” samples) versus those containing just three polyp biopsies pooled from the same coral fragment (“Polyp” samples). Due to logistical constraints, Polyp samples were generated only for fragments from the outbreak site and one control site (Site 3).

Approximately one year after the initial outbreak response sampling, a follow-up set of metabolomic samples (Year 2) were collected from the same sites but colonies were haphazardly selected compared to the targeted sampling of Year 1. Year 2 samples were collected exclusively from visually-healthy coral colonies as the outbreak had subsided by this time and diseased colonies from Year 1 had been destroyed by the outbreak. Year 2 metabolomic samples were processed and analyzed using the same methods as Year 1 data and used to check whether site differences in metabolome composition persisted through time or were specific to the time of sampling coinciding with the outbreak event.

### Metabolome methods

#### Metabolome sample processing

Coral fragments collected for metabolomic analysis were stored at − 20 °C in methanol for approximately six months prior to an aliquot being transferred to Metabolomics Australia. Aliquots of methanol extracts were centrifuged at maximum speed in a desktop centrifuge and transferred to HPLC vials. Untargeted LC–MS profiling analysis was carried out by Metabolomics Australia on a Sciex TripleTOF 6600 mass spectrometer fitted with a Duospray ion source (AB Sciex, Framingham, MA, USA) coupled to an Agilent 1290 HPLC system (Agilent Technologies, Santa Clara, CA, USA) comprised of a vacuum degasser, binary pump, thermostated auto-sampler and column oven. Methodology was adapted from Tsugawa et al. (2019) and chromatographic conditions were maintained. In brief, the analytical conditions were: Acquity bridged ethyl hybrid C18 column (1.7 μm, 2.1 mm × 100 mm, Waters); solvent A (0.1% formic acid in H2O) and solvent B (0.1% formic acid in acetonitrile); solvent gradient: 99.5% solvent A/0.5% solvent B at 0 min, 99.5%A/0.5%B at 0.1 min, 20%A/80%B at 10 min, 0.5%A/99.5%B at 10.1 min, 0.5%A/99.5%B at 12.0 min, 99.5%A/0.5%B at 12.1 min, and 99.5%A/0.5%B at 15.0 min; flow rate: 0.3 ml min^−1^ at 0 min, 0.3 ml min^−1^ at 10 min, 0.4 ml min^−1^ at 10.1 min, 0.4 ml min^−1^ at 14.4 min, and 0.3 ml min^−1^ at 14.5 min; column temperature: 40 °C; MS settings: positive mode with data collected in m/z range 70–1700 (TOF MS); curtain gas: 25 psi; GS1: 20 psi; GS2: 15 psi; ion spray voltage 5 kV; source temperature: 450 °C; accumulation time 25 ms; SWATH MS2 spectra were acquired in 25 m/z increments from 100 to 1000 m/z (35 isolation windows) and accumulation time of 25 ms. The collision energy for each window was set to 30 with a spread of 10. Feature extraction was conducted in MS-DIAL (v3.96; http://prime.psc.riken.jp/Metabolomics_Software/MS-DIAL/) and annotated using an in-house library of chemical standards and the ‘Plant Specialized Metabolome Annotation’ database (PlaSMA).

### Microbiome methods

#### Holobiont microbiome protocol

Genomic DNA was extracted from ~ 0.3 (0.2–0.6) g of preserved coral host material, containing multiple polyps and coenosarc tissue with the underlying skeleton, using the QIAGEN QIAamp DNA Mini Kit following protocols outlined in Greene et al. (2020) ^52^. For every 23 samples of genomic DNA extracted, a no-template negative control of DNA-free ultrapure water was included to identify possible contaminants introduced during the DNA extraction process. Positive controls were created by pooling approximately 2 μl of extraction elution from all 60 visually-healthy coral samples. All genomic DNA extractions, extraction negative controls, and pooled positive controls were included in PCR library preparation. The V1–V3 region of the small subunit ribosomal RNA gene 16S was targeted using genomic template primers 27f.-519r and for sequencing bacterial assemblages using the Illumina MiSeq platform (V3 chemistry, 300-bp paired-end reads) at the Ramaciotti Centre for Genomics (UNSW Sydney, Australia). Per the specifications of the sequencing facility DNA concentrations were determined using a NanoDrop (Thermo Scientific™ NanoDrop 2000) and samples over 25 ng/μl were diluted with distilled water (Invitrogen UltraPure™ DNAse/RNAse-free distilled water) to a DNA concentration between 8 and 20 ng/μl.

#### Polyp microbiome protocol

Genomic DNA was extracted from decalcified coral fragments using 1 mm diameter biopsies centered on individual coral polyps. In fragments with no evidence of disease (DH or HH) biopsies were collected randomly across visually-healthy polyps (Fig. 1, upper black circles). In fragments with evidence of disease (DD) biopsies were collected from polyps at the lesion border (Fig. 1, lower black circles). In all tissue types three biopsies were collected from each coral fragment and pooled prior to DNA extraction using the Ambion RecoverAll™ Total Nucleic Acid Isolation kit and bead maceration. A total of 82 samples from the outbreak site and Control Site 3, including negative and pooled positive controls, were sampled using polyp biopsy protocols. Polyp samples were sequenced using the same protocol and sequencing facility as Holobiont samples but on a separate sequencing run. Due to logistical constraints at the time of sample processing no biopsies were performed on fragments collected from control site 2.

### Statistical analysis—microbiome

All statistical tests were applied equally for the holobiont and polyp biopsy sample sets. We utilized the QIIME2 ^53^ bioinformatics and sequence processing pipeline outlined in Greene et al. (2020) supplementary materials with the addition of several modifications ^52^. The maximum number of expected denoising errors (parameter EE) in the reverse direction was increased to five from the QIIME2 default of two. Secondly, as our samples spanned two sequencing runs on the Illumina MiSeq platform, each run was processed independently through the step of denoising with DADA2 ^54^ and then joined for amplicon sequence variant (ASV) taxonomy assignment. ASVs were assigned taxonomy using a naïve Bayesian classifier trained on the Greengenes 16S reference database. Chimeras were removed using the DADA2 implementation in QIIME2. Final estimates of ASV abundances in each sample, ASV taxonomy data, and metadata for each sample were imported into the software R for statistical analysis, primarily using the *phyloseq* ^55^ and vegan (https://github.com/vegandevs/vegan) R packages.

The R package *decontam* ^56^ was used to identify and remove suspected contaminant ASVs more prevalent in negative controls (DNA extraction blanks) than in holobiont coral tissue samples (method = “prevalence”). After removal of contaminants, all ASVs assigned with undefined Kingdom, undefined Phylum, of class Chloroplast, or of family Mitochondria were removed. Any ASV with fewer than ten occurrences across all holobiont or polyp samples was removed. Lastly, all negative control samples and pooled control samples were removed.

In an effort to minimize sample loss in each dataset, read depth was subsampled to 30,000 reads among holobiont samples and 10,000 reads among polyp samples using the *rarefy_even_depth()* function in *phyloseq* with any samples falling below this cutoff discarded*.* Where appropriate for analysis techniques ASV absolute abundance was transformed to relative abundance using the transform_sample_counts() function in *phyloseq*. Estimates of ASV richness and Shannon diversity were calculated using the *estimate_richness()* function in *phyloseq*, and the evenness of microbial communities was calculated using the *evenness()* function from the *microbiome* package. Whenever compositional differences among samples were assessed at the family or phylum level, ASV absolute abundance data were agglomerated using the *tax_glom()* function in *phyloseq* and relative abundances of each group recalculated. Plots were generated using the R package *ggplot2* and plotting parameters associated with the parent function *ggplot()* ^57^*.* Unless otherwise stated, all analyses were carried out with ASV-level relative abundance estimates and statistical methods are identical in both the holobiont and polyp datasets. Highly-prevalent “core” ASVs across all tissue types within each sampling protocol were assessed using a 75% prevalence threshold and ASV-level data.

Unless otherwise stated, all statistical tests were performed on the complete pool of samples across all available sites. Broad patterns of dispersion and distance among tissue types were investigated visually using non-metric dimensional scaling via the *vegan* function *ordinate()*. Exploratory comparisons of diversity metrics across tissue types (DD vs DH vs HH) were made via the *phyloseq* function *plot_richness()*, followed by recalculation of diversity metrics using the phyloseq function *estimate richness()* and significance testing across tissue types and sampling locations using paired Wilcoxon rank sum tests using the *vegan* function *paired.wilcox.test()*. Dissimilarity of ASV relative abundance among sample types was calculated as Bray–Curtis distance using the *distance()* function in *vegan.* Tests for equal dispersion across tissue types, an assumption of PERMANOVA and other analyses, were carried out using the *betadisp()* function in *vegan.* If dispersion was not significantly different among sample types, a PERMANOVA analysis was performed using the function *adonis2()* in *vegan* to determine if microbial composition of tissue types were significantly different. Alternatively, if dispersion was found to be significantly different, ANOSIM analysis with reduced assumptions of equal variance was completed using the *anosim()* function in *vegan* with the grouping factor set to tissue type and strata set to sample site. An initial PERMANOVA of only HH samples from all sites was used to determine if the baseline “healthy” (HH) microbiome of sampling locations differed and require a site effect included in later PERMANOVA tests of specific tissue types across samples (DD vs DH vs HH). The initial PERMANOVA test took the form:

$${\text{adonis2}}({\text{AllSites}}\_{\text{HHSamples}}\_{\text{DistanceMatrix}}\sim {\text{ Site}},{\text{ by }} = "{\text{terms}}",{\text{ permutations }} = 1000)$$

While later PERMANOVA tests comparing different tissue types, for example DH vs HH at the outbreak site, took the form:

$${\text{adonis2}}({\text{Site1}}\_{\text{DH}}\_{\text{HH}}\_{\text{DistanceMatrix}} \sim {\text{TissueType}}, {\text{ by }} = \, "{\text{terms}}"{},{\text{ permutations }} = { 1}000)$$

PERMANOVA analysis was conducted using Bray-Curtis dissimilarity to determine if dissimilarity among tissue types within the holobiont and biopsy sample sets was significant and additional ANOSIM analysis was conducted on both datasets to confirm results. An additional ANOSIM was performed only among samples from apparently healthy colonies at all three sites to determine if significant, site-level differences in microbial composition existed.

Following assessment for broad microbial dissimilarity among tissue types or sampling locations, the package *EdgeR* ^58^ and function *phyloseq_to_edgeR()* was used to conduct binary tests to determine differential abundance of individual ASVs between tissues from diseased colonies (DD or DH) and tissues from apparently healthy colonies at the outbreak site or at control sites (HH). In all cases these tests were carried out using an ASV abundance variance threshold of 1e−5 to remove highly invariant ASVs from consideration, and a false discovery rate cutoff of 0.001 to limit false positives for differentially abundant ASVs. Paired binary tests were conducted between complete pairs of DD and DH tissues at the outbreak site to fully utilize the paired nature of the original sampling design and determine intra-colony differentially abundant ASVs indicative of diseased or healthy fragments.

After the completion of analysis on both the holobiont and polyp biopsy sample sets, results were compared to determine if similar patterns of variation were observed despite the large difference in sampling scale between these sampling protocols. Due to potential sampling bias induced from multiple sequencing runs, different sample rarefaction between holobiont or polyp sample sets, or the different tissue compartments sampled by these methods, we generally refrained from making direct comparisons of the microbiomes detected by these different sampling protocols. Instead, throughout these analyses we strived to test for patterns that were robust to these potentially biasing factors and were widely evident regardless of sampling protocol used (holobiont, polyp, or metabolome).

### Statistical analysis: metabolome

As a sparse, feature-table dataset with similar format to microbial data but different in its assumptions, metabolomic data from Year 1 and Year 2 was brought into R using the *phyloseq* package to relate sample metabolomic content to sample metadata with taxonomy and phylogenetic *phyloseq* elements set to *null*. Year 1 metabolomic data consisted of 4173 features detected across 99 samples and Year 2 metabolomic data consisted of 4605 features detected across 51 follow-up samples. As expected, metabolomic data consisting of peak intensity values for each feature was highly zero-inflated and required a log-transformation in addition to an offset of 1 to avoid undefined values for features with a detected peak intensity of zero. To inspect for persistent site effects, Bray–Curtis dissimilarity was calculated among all Year 1 Metabolome-HH samples and ordinated by site using the function *ordinate()* in the R package *vegan.* This process was repeated for Year 2 data and inter-site patterns compared. Additional PERMANOVA tests were performed on HH-only samples from the Year 1 dataset and the Year 2 follow-up samples of visually-healthy corals to verify that healthy coral metabolomes varied by site in both datasets. Following this, differing metabolome compositions were inspected among tissue types within Year 1 data. Year 1 data including all tissue types were ordinated by sample site and tissue type using the *ordinate()* function in the R package *vegan*. Due to the overdispersion of Metabolome-DD samples, a secondary ordination was completed without these samples to determine if any relationship among Year 1 DH and HH samples was more evident in their absence. After inspecting visual patterns of variance, a test for dispersion using the *vegan* function *betadisp()* confirmed significant differences in the dispersion among metabolite sample types, indicating potentially unreliable performance of PERMANOVA tests. ANOSIM analysis using the *anosim()* function in *vegan* was performed on Bray–Curtis dissimilarity values including a grouping factor for tissue type (DD vs DH vs HH) and a strata for sample site.

### Supplementary Information

Supplementary Information 1.

## Data Availability

All data, including sequencing data and analysis code files, are freely available via an open-access Open Science Framework repository located at: https://osf.io/gpyz2/.
