# Supplementary material for: Spatial extent of dysbiosis in the branching coral Pocillopora damicornis during an acute disease outbreak
Source: Sci Rep. 2023 Oct 2;13:16522. doi: 10.1038/s41598-023-43490-3 (PMC10545779; doi:10.1038/s41598-023-43490-3)
Supplement: Supplementary file 1 — Supplementary Information 1. [file 41598_2023_43490_MOESM1_ESM.docx]

**Spatial extent of dysbiosis in the branching coral *Pocillopora damicornis* during an acute disease outbreak**

Austin Greene^1,2,3,^*, Tess Moriarty^4^, William Leggatt^4^, Tracy D. Ainsworth^5^, Megan J. Donahue^1,2^, Laurie Raymundo^6^

^1^ University of Hawai‘i at Mānoa, Honolulu, USA

^2^ Hawai‘i Institute of Marine Biology, Kāne‘ohe, HI

^3^ Woods Hole Oceanographic Institution, Woods Hole, USA

^4^ University of Newcastle, Ourimbah, Australia

^5^ University of New South Wales, Sydney, Australia

^6^ University of Guam Marine Laboratory, Guam, USA

* [AustinLG@Hawaii.edu](mailto:AustinLG@Hawaii.edu)

All authors contributed equally to this work.

**Corresponding author email:**  [austinlg@hawaii.edu](mailto:austinlg@hawaii.edu)

**This PDF file includes:**

Table S1 to S3

Figures S1 to S3

**Other supporting materials for this manuscript include the following:**

**Note:** Datasets S1 to S3 include sequence data and analysis code files, described below, and are made available via an open-access repository at the Open Science Framework: <https://osf.io/gpyz2/>

Dataset_S1_PolypAnalysis.zip includes all data and code files related to the analysis of coral samples assessed as polyp biopsies in this manuscript.

Dataset_S2_HolobiontAnalysis.zip includes all data and code files related to coral samples assessed as holobiont samples in this manuscript.

Dataset_S3_MetabolomicsAnalysis.zip includes all data and code files related to coral samples assessed using mass spectrometry in this manuscript.

CoreTaxa.csv includes a listing of all taxa identified at 75% prevalence or higher in DD, DH, HH (sites pooled) tissue types for both polyp and holobiont sampling methods. Representative sequences tied to each ASV identifier in this dataset are available in Datasets S1 and S2 for polyp and holobiont samples, respectively.

**Table S1.** Sequencing read depth for across all polyp biopsy and holobiont samples prior to rarefaction.

| **Sample type** | **Min** | **25%** | **50%** | **Mean** | **75%** | **Max** |
| --- | --- | --- | --- | --- | --- | --- |
| **Holobiont** | 572 | 64622 | 80042 | 75222 | 95854 | 151130 |
| **Polyp** | 2222 | 25915 | 42066 | 43804 | 55913 | 127825 |

**Table S2.** Results of pairwise Wilcoxon rank sum tests assessing differences in microbial diversity and richness for differing tissue types sampled at the outbreak site and one control site using polyp biopsies. Due to logistical constrains fragments from Site 2 were not processed for biopsies.

P-values shown are adjusted for false discovery rates and significant results are in bold.

| **Polyp – Microbial Shannon Diversity** | | | |
| --- | --- | --- | --- |
|  | Outbreak-DD | Outbreak-DH | Outbreak-HH |
| Outbreak-DH | **1.9e-6** |  |  |
| Outbreak-HH  Site 3-HH | **3.1e-6**  **3.4e-8** | 0.24  **2e-3** | 0.18 |
| **Polyp – Microbial Richness** | | | |
|  | Outbreak-DD | Outbreak-DH | Outbreak-HH |
| Outbreak-DH | **1.8e-6** |  |  |
| Outbreak-HH  Site 3-HH | **3.5e-8**  **1.8e-6** | 0.66  0.66 | 0.84 |

**Table S3.** Results of pairwise Wilcoxon rank sum tests assessing differences in microbial diversity and richness for differing tissue types sampled at the outbreak site and two control sites using holobiont sampling methods. P-values shown are adjusted for false discovery rates and significant results are in bold.

| **Holobiont – Microbial Shannon Diversity** | | | | |
| --- | --- | --- | --- | --- |
|  | Outbreak-DD | Outbreak-DH | Outbreak-HH | Site 2-HH |
| Outbreak-DH | 0.30 |  |  |  |
| Outbreak-HH | 0.25 | 0.94 |  |  |
| Site 2-HH | **0.01** | **4e-3** | **4e-3** |  |
| Site 3-HH | 0.15 | 0.16 | 0.15 | 0.25 |
| **Holobiont – Microbial Richness** | | | | |
|  | Outbreak-DD | Outbreak-DH | Outbreak-HH | Site 2-HH |
| Outbreak-DH | **0.04** |  |  |  |
| Outbreak-HH | **3e-3** | 0.22 |  |  |
| Site 2-HH | **3e-4** | **1e-3** | **2e-3** |  |
| Site 3-HH | **3e-3** | 0.06 | 0.19 | 0.32 |


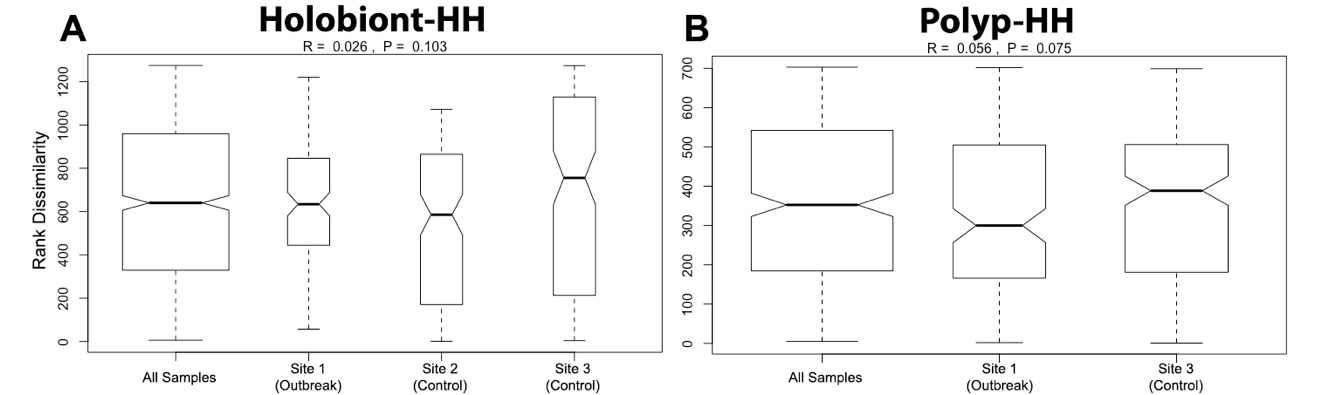


**Figure S1.** Results of analysis of similarity (ANOSIM) indicating no significant differences in microbial communities between visibly-healthy coral colonies sampled using either (A) holobiont or (B) polyp biopsy sampling methods.

**
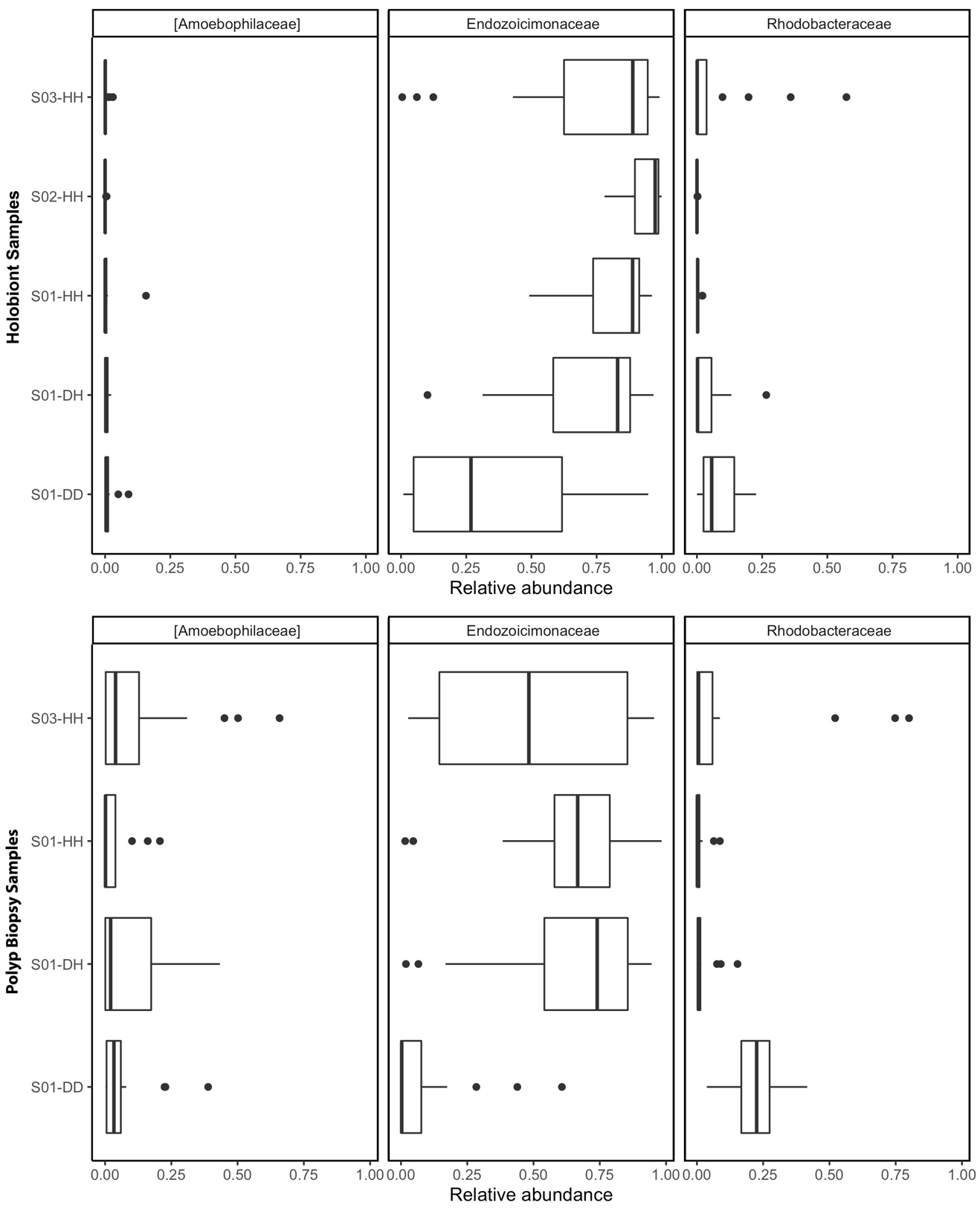
**

**Figure S2.** Relative abundance of *Candidatus: Amoebophilaceae, Endozoicomonaceae and Rhodobacteraceae* bacterial taxa across coral samples for both holobiont (top) and polyp biopsy sample collection methods (bottom).


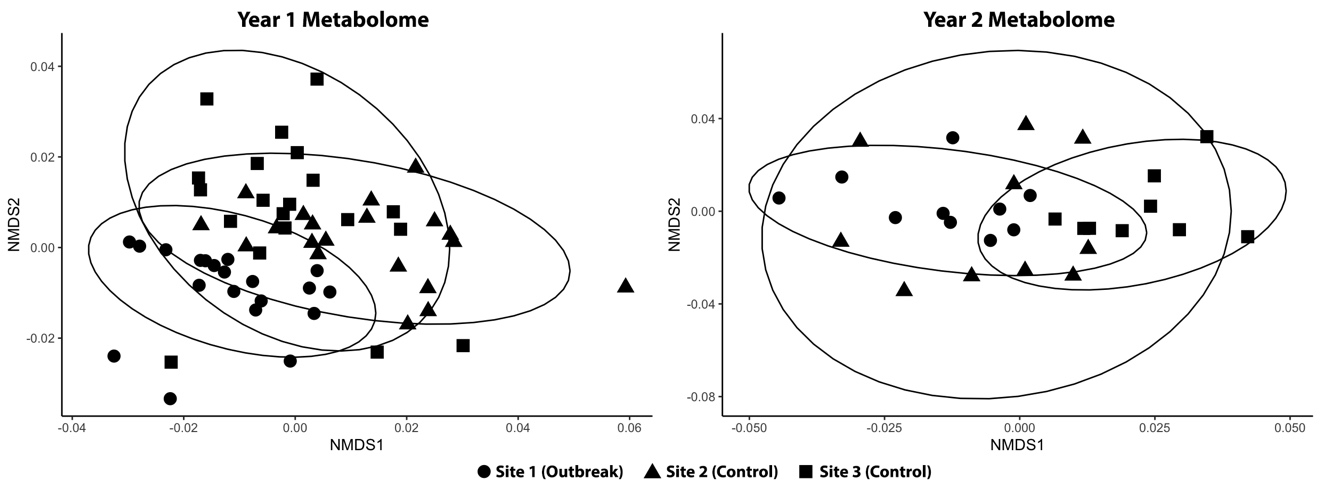


**Figure S3.** Metabolomes of visually-healthy corals maintain significant site differences between the time of the outbreak (Year 2) and one year following (Year 2). Note that haphazard sampling of corals in Year 2 means overlap between Year 1 and Year 2 sample sets is possible but unlikely.
